# Reducing butter firmness with chemically esterified butter oil

**DOI:** 10.3168/jdsc.2024-0597

**Published:** 2024-07-26

**Authors:** Talia Katz, Shoshana Ginsburg, Rafael Jimenez-Flores

**Affiliations:** Department of Food Science and Technology, The Ohio State University, Columbus, OH 43210

## Abstract

In a consumer-centric environment, food products are created and focused on the consumer's experience and desires. One important food product used around the world is butter. Consumers expect that when they take their butter out of the refrigerator, it will be able to spread nicely and evenly; however, with conventional butter, which does not include any added vegetable oils, achieving a soft, spreadable butter is rare. This work seeks to contribute to the pool of knowledge regarding the role of catalysts in effecting butter spreadability. To meet consumer expectations, utilizing catalysts in the esterification process with butter oil has proven successful in changing the lipid structure, enabling the possibility of a softer butter. The comparison of utilizing no catalysts and 2 different catalysts, potassium hydroxide and calcium hydroxide, during the chemical esterification process showed that the use of potassium hydroxide catalyst resulted in the least firm and lowest solid fat content butter. These findings demonstrate the potential for industrial use of esterified butter oil using potassium hydroxide in commercial butter.

## Highlights

• It is possible to add a small fraction of transesterified butter oil to regular butter to increase spreadability.
• The present method does not require plant oils as nondairy sources of fat.
• Scale-up or commercialization of the process would require an optimal catalyzer for optimal efficiency.
• Potassium hydroxide, which is not a usual catalyzer, yields high efficiency for the transesterification reaction.

## Graphical Abstract

**Figure:**
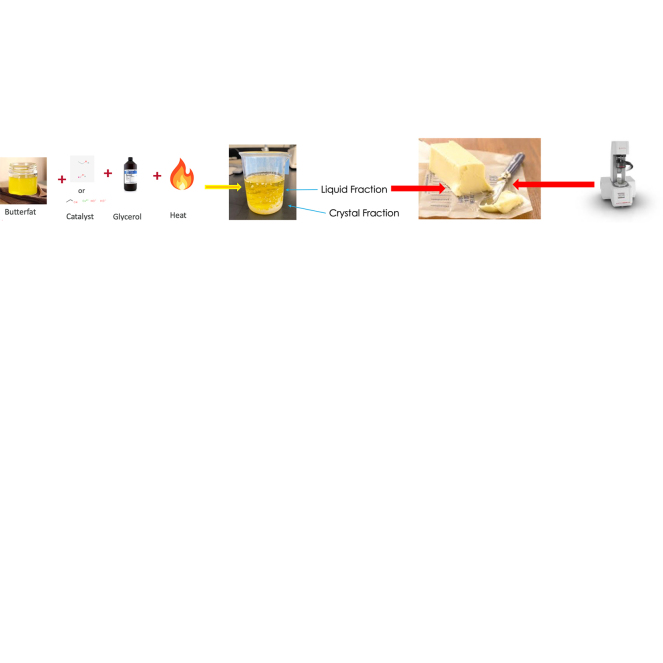
**Summary:** The public currently desires to purchase butter that is spreadable right out of the refrigerator but also to minimize additives, even plant oils. One commercial practice is to add plant oils to make the butter softer, which results in a butter that, if it stays out of the refrigerator for a long time or on a hot day, becomes too soft. One way to achieve the desired result is by transesterification of butter oil, which, due to more active reaction kinetics of short-chain fatty acids, results in a higher proportion of noncrystallizable triglycerides. This article focuses on catalyst comparison for an efficient reaction. This article contributes to our knowledge in determining that potassium hydroxide is more efficient than calcium hydroxide in generating a softer butter.

Butter serves as a key product in the dairy industry as well as an ingredient in many other food applications. Butter is made from milk by a churning process that reverses the emulsion properties of milk to be that of water in oil, where the resulting products is at least 80% fat to meet the legal definition of butter (Bylund, 2015). Anhydrous milk fat (**AMF**) is the fat product from milk after the complete removal of water and nonfat solids, and AMF ultimately makes up much of the lipid basis for butter (Bylund, 2015). Because AMF makes up such a large component of butter, seasonal and regional variations in the fat in the milk have an effect on how soft the butter is. Due to the lipid composition of anhydrous milk fat and the resulting crystallization behavior of lipids, summer AMF is softer due to fatty acid composition having a higher concentration of short-chain triacylglycerides (**TAG**) compared with winter AMF, which has more saturated and longer chain TAG (Shi et al., 2001). While seasonal variations can have effects on the TAG profile of AMF, additional methods have been employed to alter the lipid profile of AMF with the intent to produce softer butter. These methods include transesterification of milk fat.

Enzymatic interesterification is a technique that has been used and is often employed for spreads such as margarine and shortenings (Shin et al., 2010). Esterification can be used to rearrange distribution and positioning of fatty acids on the glycerol backbone, providing the possibility for shorter TAG (Shin et al., 2010). Although enzymatic esterification can be used, the process requires a great length of time, whereas the investigation of the chemical esterification process can greatly reduce the amount of time needed (Ginsburg and Jiménez-Flores, 2024). The use of various chemical catalysts has been employed to fully grasp the influence that chemical esterification has on the TAG development and its applications for increasing softness of AMF. Chemical esterification has been proven to form partial glycerides, mono- (**MAG**), and diacylglycerides (**DAG**), in the butter oil, which in turn reduces the melting temperature and storage modulus of the crystals (Ginsburg and Jiménez-Flores, 2024). This study investigates the incorporation of the products from the transesterification of butter oil by adding them into cream during the churning process and then analyzing the firmness of the complete butter product.

Butter was initially obtained from The Ohio State's University dairy pilot plant and chemicals and reagents were purchased from Fisher Scientific. Butter oil was extracted from the butter by heating it in a 65°C water bath and separating the butter oil from the aqueous butter serum, followed by careful decanting and particle filtration through filter paper of the butter oil. Butter oil was kept at room temperature until it was used for the esterification reaction. A solution of 10% (wt/wt) glycerol, 1% catalyst (wt/wt), and 89% (wt/wt) butter oil was made and mixed using a Caframo laboratory stand mixer at 750 rpm for 5 min. The 2 catalysts used were 2 *N* potassium hydroxide (KOH) in ethanol and 2 *N* calcium hydroxide [Ca(OH)_2_] in ethanol. Solutions were heated to 200°C for 2 h in a laboratory oven. After the reaction was complete, the butter oil layer was separated from the glycerol fraction and placed in a 30°C incubator for 24 h to allow for high melting crystal formation in the butter oil. After 24 h, the esterified butter oil was brought to 12°C and kept at that temperature until it was used in the butter-making process. A nonesterified butter oil was exposed to the same conditions without the addition of glycerol and a catalyst for a blank sample.

Heavy whipping cream was purchased from a local grocery store and stored at refrigeration temperature until used for butter production. Cream was added to a KitchenAid stand mixer and churned using the whisk attachment. Once butter grain formation was visible, 5% wt/wt of esterified butter oil sample was added to the grains and the paddle attachment was used to finish the churning process. The buttermilk was drained and separated from the butter. The butter was pressed into a log shape and then a cork borer was used to transfer samples into 5-mL syringes. The plunger was removed, and the sample from the cork borer was inserted into the syringe, filling approximately 4 mL. The plunger was placed back in, fully immobilized, to store at 4°C overnight.

The TA.XTplusC texture analyzer (Stable Micro Systems, Surrey, UK) and Exponent Connect software was used to measure the compression force to extrude the butter out of the syringe moving at a rate of 1 mm/s for a total distance of 50 mm to extrude the complete sample from the syringe. The texture analyzer was calibrated for both height and force (5 kg weight) before analyzing the butter samples. Data were acquired at a rate of 500 points per second with a typical test time of 150 s. Samples were kept at 4°C until testing was performed. Triplicates were completed for each butter treatment created.

Solid fat content (**SFC**) of the butter samples was measured using time domain nuclear magnetic resonance (**NMR**; Bruker Corporation, Billerica, MA) with the *mini*Spec20 NMR. Samples were placed in a 10-mm NMR tube and measured 1 d after being stored at 4°C. After calibrating the NMR with 0.0%, 31.1%, and 73.1% reference standards, the SFC of the butters were measured at room temperature. Measurements of each sample were performed in triplicates.

Measurements from the texture analyzer reported a significant reduction in firmness in the butter that had the addition of KOH-catalyzed esterified butter oil (*P* < 0.05). As seen in Figure 1, the control sample (butter with no butter oil addition) was not significantly different in firmness compared with the butter samples with the blank and Ca(OH)_2_-esterified butter oil. The Ca(OH)_2_ butter was the firmest sample (127.61 N); however, it was not significantly different in firmness compared with the control butter (125.84 N). Butters with butter oil esterified with KOH as a catalyst and blank butter oil were significantly (*P* < 0.05) less firm compared with Ca(OH)_2_ butter. Reduction in firmness for the extruder force, seen in the KOH butter samples, has been shown to correlate well to spreadability of butter, finding less firm butters to be more spreadable (Lewis, 2023). This reduction in firmness due to the addition of the esterified butter oil samples may be due to the addition of MAG and DAG that formed during the esterification reaction. Ginsburg and Jiménez-Flores (2024) demonstrate the production of MAG and DAG, in different concentrations depending on the catalyst used, from the esterification reaction. Mono- and diacylglycerols have been shown to disrupt and alter triacylglyceride crystal formation and growth (Cholakova and Denkov, 2024). The disruption in crystal formation may lead to differences in crystal sizes and thus fewer and weaker crystal formation. Ginsburg and Jiménez-Flores (2024) demonstrated that when esterifying butter oil, the butter oil crystals varied in melting temperature and enthalpy compared with blank butter oil, additionally when using KOH as a catalyst to form crystals that melt at lower temperatures and require less energy to melt them. Using Ca(OH)_2_ as a catalyst has also been found to reduce the melting and enthalpy of butter oil crystals, but the decrease is not as large compared with using KOH as a catalyst (Ginsburg and Jiménez-Flores, 2024). Moreover, as seen in Figure 2, the SFC of the butters with esterified butter oil added to them are significantly (*P* < 0.05) lower compared with the control butter. Adding esterified butter with KOH as a catalyst decreased the SFC by about 3%, where the control's SFC is 41.78% and KOH's SFC is 38.35%. These findings suggest that the crystals formed with the incorporation of MAG and DAG in the KOH-esterified butter oil increase the amount of amorphous material in the overall butter and thus produces a softer, more spreadable butter. The concentration of MAG and DAG added to TAG influences the overall effect they have on crystallization (Sato et al., 2013). Using KOH as a catalyst forms more partial glycerides, and particularly more DAG compared with using Ca(OH)_2_ as a catalyst possibly due to its lower energy of ionization (Brown, 2012; Ginsburg and Jiménez-Flores, 2024). The differences in concentration of MAG and DAG formation when using different catalysts therefore influence the crystal interactions within the butter, changing its firmness.

**Figure 1:**
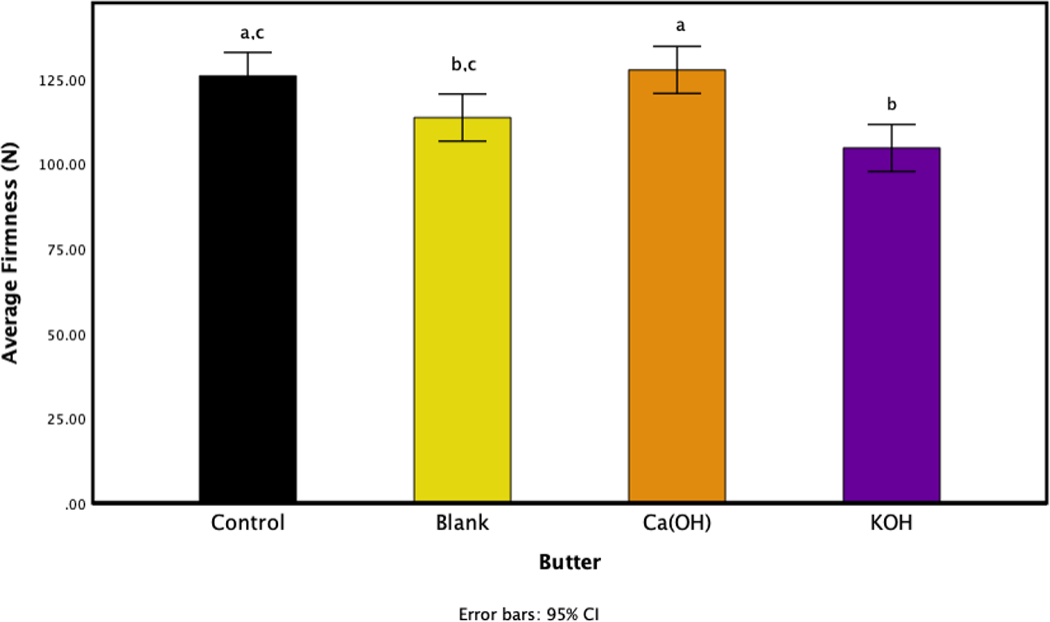
Average firmness of butter samples using forward extrusion texture analyzer control (black), addition of 5% blank butter oil (yellow), addition of 5% butter oil esterified with Ca(OH)_2_ as a catalyst (orange), and addition of 5% butter oil esterified with KOH as a catalyst (purple). Bars represent standard deviations of means, and different letters represent significance among samples (*P* < 0.05).

**Figure 2:**
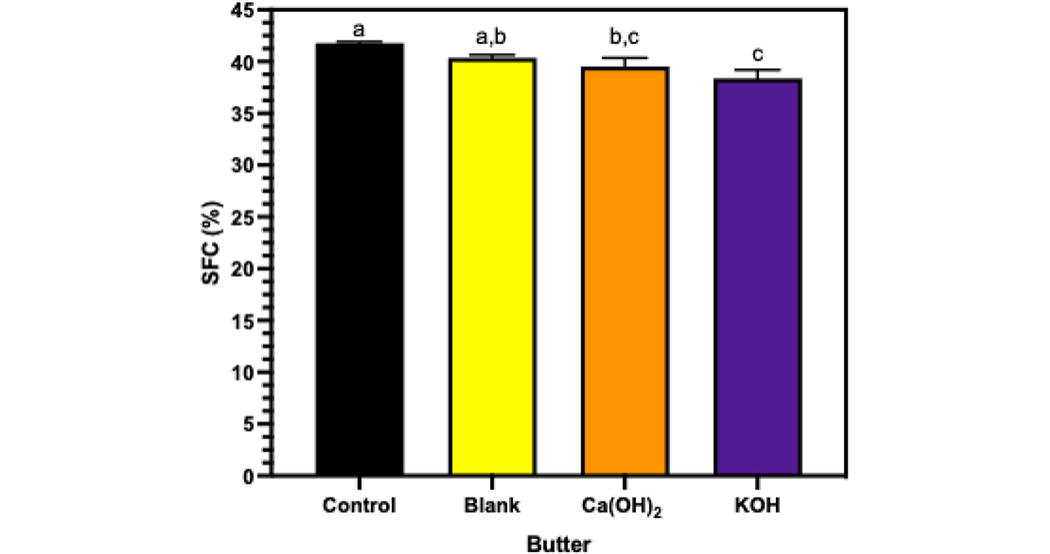
Solid fat content (SFC) of butter samples, control (black), addition of 5% blank butter oil (yellow), addition of 5% butter oil esterified with Ca(OH)_2_ as a catalyst (orange), and addition of 5% butter oil esterified with KOH as a catalyst (purple). Bars represent standard deviations of means, and different letters represent significance among samples (*P* < 0.05).

Data collected regarding both firmness and solid fat content found a 20% reduction in firmness in the KOH-esterified butter compared with control butter. Additionally, the solid fat content for KOH-esterified butter was reduced by 3%. We believe this reduction of firmness and solid fat content for KOH-esterified butter will correlate to a softer and more spreadable butter, and therefore can be used to produce a more desirable product.
